# Comparison of the Separation Performances of *Cinchona* Alkaloid-Based Zwitterionic Stationary Phases in the Enantioseparation of *β*^2^- and *β*^3^-Amino Acids

**DOI:** 10.3390/molecules20010070

**Published:** 2014-12-23

**Authors:** István Ilisz, Nóra Grecsó, Aleksandra Misicka, Dagmara Tymecka, László Lázár, Wolfgang Lindner, Antal Péter

**Affiliations:** 1 Department of Inorganic and Analytical Chemistry, University of Szeged, Dóm tér 7, H-6720 Szeged, Hungary; E-Mail: ilisz@chem.u-szeged.hu (I.I.); grecso.nora@pharm.u-szeged.hu (N.G.); 2 Institute of Pharmaceutical Chemistry, University of Szeged, Eötvös utca 6, H-6720 Szeged, Hungary; E-Mail: lazar@pharm.u-szeged.hu; 3 Department of Chemistry, University of Warsaw, Pasteura str. 1, 02-093 Warsaw, Poland; E-Mails: misicka@chem.uw.edu.pl (A.M.); dulok@chem.uw.edu.pl (D.T.); 4 Department of Analytical Chemistry, University of Vienna, Währinger Strasse 38, 1090 Vienna, Austria; E-Mail: wolfgang.lindner@univie.ac.at

**Keywords:** column liquid chromatography, *β*^2^-amino acids, *β*^3^-amino acids, *Cinchona* alkaloid-based chiral stationary phases

## Abstract

The enantiomers of twelve unusual *β*^2^- and *β*^3^-homoamino acids containing the same side-chains were separated on chiral stationary phases containing a quinine- or quinidine-based zwitterionic ion-exchanger as chiral selector. The effects of the mobile phase composition, the nature and concentration of the acid and base additives and temperature on the separations were investigated. The changes in standard enthalpy, ∆(∆*H*°), entropy, ∆(∆*S*°), and free energy, ∆(∆*G*°), were calculated from the linear van’t Hoff plots derived from the ln *α vs.* *1/T* curves in the studied temperature range (10–50 °C). The values of the thermodynamic parameters depended on the nature of the selectors, the structures of the analytes, and the positions of the substituents on the analytes. A comparison of the zwitterionic stationary phases revealed that the quinidine-based ZWIX(−)™ column exhibited much better selectivity for both *β*^2^- and *β*^3^-amino acids than the quinine-based ZWIX(+)™ column, and the separation performances of both the ZWIX(+)™ and ZWIX(−)™ columns were better for *β*^2^*-*amino acids. The elution sequence was determined in some cases and was observed to be *R <* *S* and *S* < *R* on the ZWIX(+)™ and ZWIX(−)™ columns, respectively.

## 1. Introduction

In view of their diverse applications in various areas of organic and medicinal chemistry, *β*-amino acids have received increased attention in recent decades. They are present in numerous natural or synthetic bioactive molecules (e.g., taxane derivatives, sitagliptin, *β*-lactam antibiotics, *etc.*). *β*-Amino acids are applied in the synthesis of heterocyclic derivatives with wide structural diversity, and a rapidly growing class of oligomeric peptides with considerable biological and catalytic properties [1,2,3,4,5]. The newly developed enantioselective syntheses of *β*-amino acids [5,6,7,8] require analytical methods for a check on the enantiopurity of the final products.

The separation and identification of *β*-amino acid enantiomers have mainly been performed by indirect [9,10] and direct high-performance liquid chromatographic (HPLC) methods [11,12,13,14,15,16,17,18,19,20,21,22,23,24,25,26,27]. Several types of chiral stationary phases (CSPs) have been applied for the enantioseparation of *β*-amino acids. Macrocyclic glycopeptide-based CSPs were utilized by D’Acquarica *et al.* [12] and Péter *et al.* [13,14]. Since the introduction of chiral crown ethers as CSPs by Cram *et al.* [15], a (+)-(18-crown-6)-2,3,11,12-tetracarboxylic acid-based CSP has been successfully employed for the enantioseparation of *β*-amino acids by Hyun *et al.*, [16,17,18,19,20,21] and Péter *et al.*, [22,23]. A chiral ligand exchange column [24] and newly developed zwitterionic *Cinchona* alkaloid-based selectors [25,26,27] were recently used for the enantioseparation of *β*-amino acids.

Enantioselective retention mechanisms are well known to be influenced by temperature. Accordingly, as demonstrated in several papers [28,29,30,31,32,33], optimization of the column temperature is essential in enantioselective HPLC separations.

The dependence of the retention of an analyte on the temperature can be expressed by the van’t Hoff equation, which may be interpreted in terms of mechanistic aspects of chiral recognition:

(1) $$lnk\prime =-\frac{\Delta H^{o}}{RT}+\frac{\Delta S^{o}}{R}+\text{ln}\phi$$

where ∆*H°* is the standard enthalpy of transfer of the solute from the mobile phase to the CSP, ∆*S°* is the standard entropy of transfer of the solute from the mobile phase to the CSP, *R* is the gas constant, *T* is the temperature in Kelvin and *ϕ* is the phase ratio of the column (the volume of the stationary phase divided by the volume of the mobile phase). If *ϕ* is constant or independently measured, ∆*H*° can be determined by using the van’t Hoff plot (if ∆*H°* is invariant with temperature) [34,35]. Unfortunately, *ϕ* is not known in most cases, and even the definition of the stationary phase volume for bonded-phase columns is doubtful. However, if both enantiomers have access to the same stationary phase volume, the ∆(∆*H*°) and ∆(∆*S*°) values for the separated enantiomers can be determined from the modified equation:

(2) $$ln\alpha =-\frac{\Delta (\Delta H^{o})}{RT}+\frac{\Delta (\Delta S^{o})}{R}$$

where *α* is the selectivity factor (*α = k*_2_*/k*_1_), ∆*(*∆*H*°) is the difference in the standard enthalpy change, and ∆*(*∆*S*°) is the difference in the standard entropy change for the two enantiomers. With this simplified approach, a plot of *R* ln *α* *vs.* 1/*T* has slope −∆(∆*H*°) and intercept ∆(∆*S*°).

For the purposes of this study, the classical van’t Hoff approach assuming only one site interaction was used. For a more realistic approach to the thermodynamic calculations, the contributions of enantioselective and non-selective sites should be distinguished. This can be achieved through the application of non-linear characterization methods [36,37].

The aim of the present work was to investigate the effectiveness of *Cinchona* alkaloid-based CSPs for the separation of a series of isobaric *β*^2^- and *β*^3^-homoamino acids. For comparison purposes, most of the separations were carried out at constant mobile phase compositions at different temperatures. The influence of specific structural features of the analytes and selectors and the effects of temperature on the retention will be discussed on the basis of the experimental data. The elution sequence was in some cases determined by spiking the racemate with an enantiomer with known absolute configuration.

## 2. Results and Discussion

**Figure 1:**
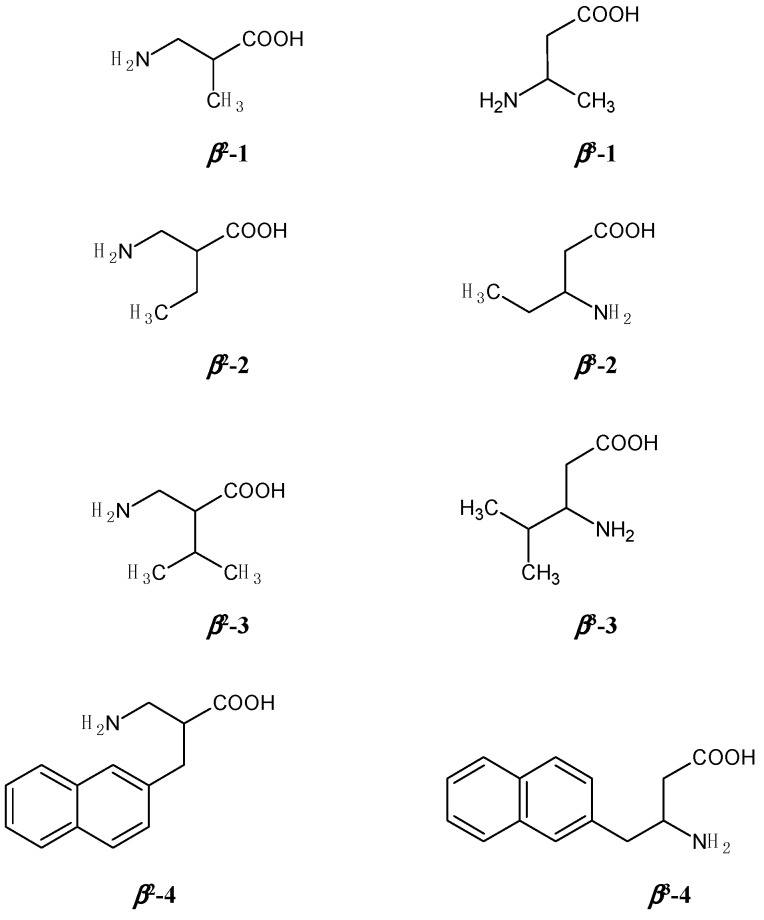

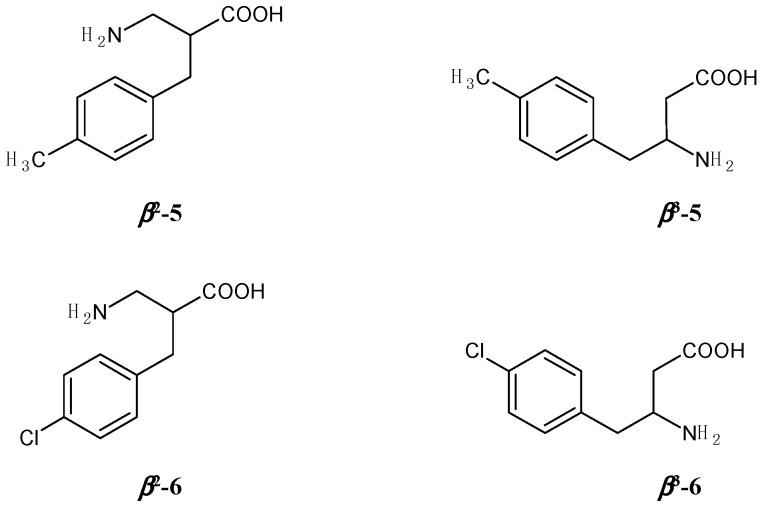
Structures of investigated *β*^2^ and *β*^3^ amino acids: 3-Amino-2-methylpropanoic acid (*β*^2^**-1**), 2-aminomethylbutanoic acid (*β*^2^-**2**), 2-aminomethyl-3-methylbutanoic acid (*β*^2^**-3**), 3-amino-2-(naphthalen-2-ylmethyl)propionic acid (*β*^2^-**4**), 3-amino-2-(4-methyl- benzyl)propionic acid (*β*^2^*-***5**) and 3-amino-2-(4-chlorobenzyl)propionic acid (*β*^2^*-***6**), 3-aminobutanoic acid (*β*^3^*-***1**), 3-aminopentanoic acid (*β*^3^-**2**), 3-amino-4-methylpentanoic acid (*β*^3^**-3**), 3-amino-4-(naphthalen-2-yl)butanonic acid (*β*^3^*-***4**), 3-amino-4-(4-methylphenyl)butanoic acid (*β*^3^**-5**), 3-amino-4-(4-chlorophenyl)butanoic acid (*β*^3^**-6**).

**Figure 2:**
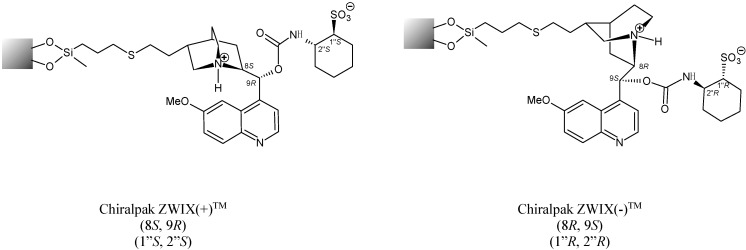
Structures of quininine- [ZWIX(+)™] and quinidine-based [ZWIX(−)™] CSPs.

The two classes of analytes, *β*^2^- and *β*^3^-homoamino acids, differ in the positions of their substituents (Figure 1). In both classes, analogues **1**, **2** and **3** bear alkyl groups, which may have different steric effects. This influences the hydrophobicity, bulkiness and rigidity of the molecules. Compounds **4**, **5** and **6** possess aromatic rings, potentially able to undergo π-π, steric/rigid or other interactions with the aromatic structure elements of the chiral selector and CSP (Figure 2).

### 2.1. Effect of the Mobile Phase Composition

The *Cinchona*-derived CSPs have often been used in non-aqueous polar ionic mode (PIM), with MeOH and MeCN as bulk solvent, together with acid and base additives [38]. The polar protic character of MeOH weakens the H-bonding interactions, while MeCN as an aprotic solvent weakens the aromatic *π*-*π* interactions and strengthens the ionic interactions. To ensure primary ionic interactions with the selector (SO), an acid and base are generally added to the non-aqueous polar organic solvents, acting as ion pair and charge-giving components of the ionizable ampholytic SO and the selectand (SA).

**Figure 3:**
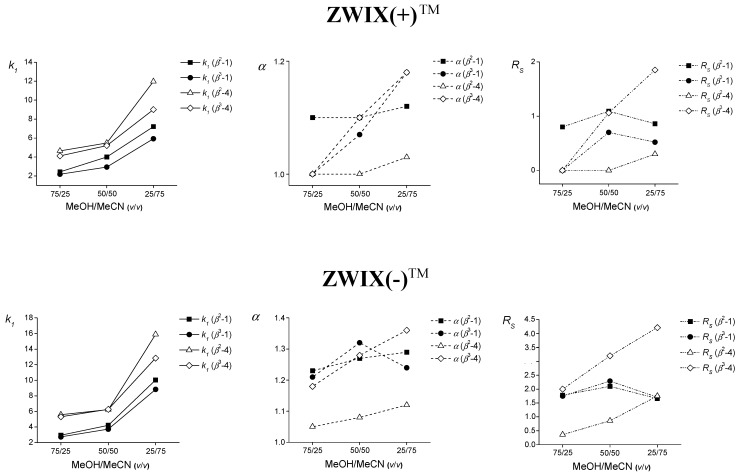
Effects of the compositions of the bulk solvents on the chromatographic parameters for compounds *β*^2^-**1**, *β*^3^-**1**, *β*^2^-**4** and *β*^3^-**4** on ZWIX(+)™ or ZWIX(−)™ CSPs. Chromatographic conditions: column, Chiralpak ZWIX(+)™ and ZWIX(−)™; mobile phase, **a**, MeOH/MeCN (75/25 *v*/*v*) containing 25 mM PRA and 50 mM AcOH, **b**, MeOH/MeCN (50/50 *v*/*v*) containing 25 mM PRA and 50 mM AcOH, **c**, MeOH/MeCN (25/75 *v/v*) containing 25 mM PRA and 50 mM AcOH **d**, MeOH/MeCN (75/25 *v*/*v*) containing 25 mM TPRA and 50 mM AcOH, **e**, MeOH/MeCN (50/50 *v*/*v*) containing 25 mM TPRA and 50 mM AcOH, **f**, MeOH/MeCN (25/75 *v/v*) containing 25 mM TPRA and 50 mM AcOH; flow rate, 0.6 mL·min^−1^; detection, 215 and 230 nm or corona detector; temperature 25 °C; symbols, *k*_1_: 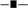: for *β*^2^**-1**, 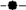: for *β*^3^**-1**, 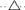: for *β*^2^**-4** and 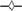: for *****β*^3^**-4**; *α*: 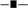: for *β*^2^**-1**, 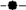: *β*^3^**-1**, 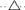: for *β*^2^**-4** and 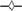: for *β*^3^**-4**; *R_S_*: 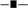: for *β*^2^**-1**, 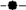: *β*^3^**-1**, 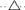: for *β*^2^-**4** and 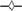: for *β*^3^**-4**.

The influence of the composition of bulk solvents on the chromatographic parameters was investigated for analytes containing an alkyl (**1**) or aryl (**4**) side-chain, on ZWIX(+)™ and ZWIX(−)™ in the presence of MeOH as protic and MeCN as aprotic solvent. The mobile phase system consisted of MeOH/MeCN, with increasing amounts of MeCN in MeOH (25%, 50% and 75%), and contained 50 mM AcOH and 25 mM propylamine (PRA) or 50 mM AcOH and 25 mM tripropylamine (TPRA), the acid-to-base ratio being kept constant at 2:1 (Figure 3 and Supplementary Material Tables S1 and S2). As in earlier studies [39,40] on both columns in a MeOH/MeCN bulk solvent system containing 50 mM AcOH and 25 mM PRA, the retention of zwitterionic amino acids increased substantially with increasing MeCN content (Figure 3). Similar results were obtained on both columns in the MeOH/MeCN eluent system containing 50 mM AcOH and 25 mM TPRA (Supplementary Material, Tables S1 and S2). This trend was observed for all the investigated analytes on both stationary phases.

As concerns the selectivity, regardless of the 2 or 3 position of the substituent, the separation factor (*α*) increased with increasing MeCN content [an exception was *β*^3^-**1** on the ZWIX(−)™ column] in the presence of PRA as base additive: the highest *α* value was obtained in MeOH/MeCN (50/50 *v/v*) as mobile phase. The effect of the bulk solvent composition on the *k* and *α* values can be summarized as follows: (*a*) an increase of the content of the aprotic, but polar MeCN probably decreases the solvation of the analytes, but also strengthens the electrostatic interactions with the (SO), leading to increased retention times; (*b*) the increase in *α* in the MeCN-rich mobile phase was probably due to the enhanced electrostatic and H-bonding interactions, promoting the chiral recognition. The higher MeOH content in the bulk solvent promotes the solvation of the analytes, thereby weakening the overall interactions of the SO with the SAs.

For the *β*-amino acids, the *R_S_* values increased considerably with increasing MeCN content. However, analytes *β*^2^-**1** and *β*^3^-**1** on both the ZWIX(+)^TM^ and ZWIX(−)^TM^ columns exhibited maximum values at MeOH/MeCN (50/50 *v/v*) bulk solvent composition (Figure 3 and Supplementary Materials Tables S1 and S2). On the basis of these results, all further experiments were carried out with a 50/50 (*v/v*) mixture of MeOH/MeCN as bulk solvent. These results indicate that, in general, MeCN as an aprotic solvent is much more attractive than MeOH as a protic solvent for the chiral recognition of *β*^2^- and *β*^3^-amino acid enantiomers on the zwitterionic CSPs.

To regulate the primary ionic interaction between the SO and SAs, the non-aqueous polar organic solvents are generally modified by the addition of acid and base. The additives greatly influence the solvation of both SOs and SAs, and the anions and cations of acid and base additives acting as charged displacers exert great effects on the elution strength of the mobile phase.

For a more detailed investigation of the effects of the nature of the acid and base additives, separations of analytes **1** and **4** were carried out with the same bulk solvent composition MeOH/MeCN (50/50 *v/v*) containing 25 mM base and 50 mM acid on both the ZWIX(+)™ and ZWIX(−)™ columns. Four different bases [PRA, TPRA, butylamine (BA) and tributylamine (TBA)] were selected, which differed in the degree and nature of their alkyl substitution. An excess of the acid component in the mobile phase (acid-to-base concentration ratio ~2) ensured that the bases were present in their protonated “ammonium-ion” form.

**Figure 4:**
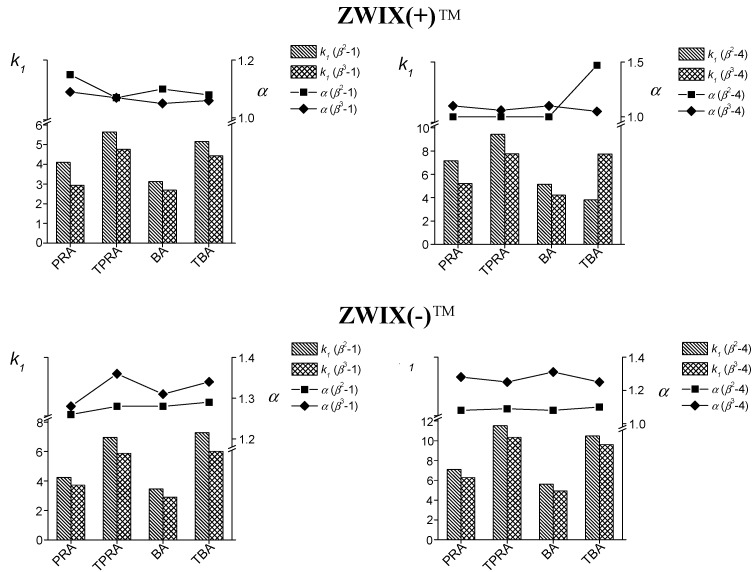
Effects of acid and base additives on the chromatographic parameters for the resolution of compounds *β*^2^-**1**, *β*^3^-**1**, *β*^2^-**4** and *β*^3^-**4** on *Cinchona* alkaloid-based CSPs. Chromatographic conditions: column, Chiralpak ZWIX(+)™ and ZWIX(−)™; mobile phase, MeOH/MeCN (50/50 *v/v*) containing **b**, 25 mM PRA and 50 mM AcOH, **e**, 25 mM TPRA and 50 mM AcOH, **g**, 25 mM BA and 50 mM AcOH, **h**, 25 mM TBA and 50 mM AcOH; flow rate, 0.6 mL·min^−1^; detection 215 and 230 nm or corona detector; temperature, 25 °C.

For all the analytes on both columns, the retention factors increased when the degree of propyl and/or butyl substitution of the *nitrogen* increased with the exception of *ß*^2^-4 (Figure 4 and Table 1 and Table 2). The observed increased retention is probably due to the reduced capacity of TBA or TPRA in their roles as counter-ions comparing to the BA or PRA, respectively. As concerns the influence of the nature of the bases on the separation factor, a slight change in *α* was observed without any general trend. On the ZWIX(+)™ CSP, *α* varied between 1.00 and 1.57, while on ZWIX(−)™ it varied between 1.08 and 1.68. The *R_S_* values on ZWIX(+)^TM^ were generally higher with PRA as base additive, while on ZWIX(−)™ this was true for TPRA. When the effects of base additives on the retention and selectivity are taken into account, the application of PRA appears favourable.

A comparison of the separation performances of the ZWIX(+)^TM^ and ZWIX(−)^TM^ CSPs at the same eluent composition, independently of the nature of the base additives reveals that the *k, α* and *R_S_* values for the *β*^2^- and *β*^3^-amino acids were generally higher on the ZWIX(−)™ CSP than on the ZWIX(+) CSP.

**Table 1:** Chromatographic data, separation factor (*k*), selectivity factor (*α*), resolution (*R_S_*) and elution sequence of *β*^2^- and *β*^3^-amino acids on ZWIX(+)™ column.

Compound	Eluent	*k*_1_	*α*	*R_S_*	*Elution sequence*
***β*^2^-1**	**g**	3.12	1.10	0.63	*-*
	**h**	5.15	1.08	0.32	*-*
***β*^3^-1**	**g**	2.69	1.05	0.43	*R* < *S*
	**h**	4.44	1.06	0.32	*R < S*
***β*^2^-2**	**b**	3.45	1.29	2.80	*-*
	**e**	5.35	1.28	1.96	-
	**g**	3.06	1.28	2.62	*-*
	**h**	5.39	1.25	1.98	*-*
***β*^3^-2**	**b**	2.85	1.13	1.37	*-*
	**e**	4.24	1.13	1.50	*-*
	**g**	2.52	1.13	1.19	*-*
	**h**	4.17	1.13	1.04	*-*
***β*^2^-3**	**b**	3.10	1.57	4.56	*R < S*
	**e**	5.09	1.45	4.00	*R < S*
	**g**	2.78	1.53	4.25	*R < S*
	**h**	5.02	1.46	3.48	*R < S*
***β*^3^-3**	**b**	2.48	1.18	1.88	*-*
	**e**	3.62	1.19	2.23	*-*
	**g**	2.21	1.18	1.60	*-*
	**h**	3.57	1.19	1.27	*-*
***β*^2^-4**	**g**	5.16	1.00	0.00	*-*
	**h**	3.81	1.47	3.27	*-*
***β*^3^-4**	**g**	4.22	1.10	0.83	*R* < *S*
	**h**	7.75	1.05	0.36	*R* < *S*
***β*^2^-5**	**b**	4.43	1.03	0.26	*-*
	**e**	6.87	1.05	0.42	*-*
	**g**	3.92	1.03	<0.20	*-*
	**h**	7.02	1.04	0.31	*-*
***β*^3^-5**	**b**	3.67	1.10	1.05	*R* < *S*
	**e**	5.60	1.07	0.79	*R* < *S*
	**g**	3.26	1.10	1.05	*R* < *S*
	**h**	5.63	1.07	0.80	*R* < *S*
***β*^2^-6**	**b**	5.08	1.02	<0.20	*-*
	**e**	8.26	1.06	0.60	*-*
	**g**	4.51	1.00	0.00	*-*
	**h**	8.39	1.05	0.36	*-*
***β*^3^-6**	**b**	4.35	1.15	1.67	*R* < *S*
	**e**	6.80	1.12	1.31	*R* < *S*
	**g**	3.82	1.14	1.47	*R* < *S*
	**h**	6.79	1.13	1.41	*R* < *S*

Notes: Chromatographic conditions: column, Chiralpak ZWIX(+)™; mobile phase, **b**, MeOH/MeCN (50/50 *v/v*) containing 25 mM PRA and 50 mM AcOH, **e**, MeOH/MeCN (50/50 *v/v*) containing 25 mM TPRA and 50 mM AcOH, **g**, MeOH/MeCN (50/50 *v/v*) containing 25 mM BA and 50 mM AcOH, **h**, MeOH/MeCN (50/50 *v/v*) containing 25 mM TBA and 50 mM AcOH; flow rate 0.6 mL·min^−1^; 215, 230 nm and corona detection.

**Table 2:** Chromatographic data, separation factor (*k*), selectivity factor (*α*), resolution (*R_S_*) and elution sequence of *β*^2^- and *β*^3^-amino acids on ZWIX(−)™ column.

Compound	Eluent	*k*_1_	*α*	*R_S_*	*Elution sequence*
***β*^2^-1**	**g**	3.46	1.28	3.39	-
	**h**	7.29	1.29	3.08	-
***β*^3^-1**	**g**	2.91	1.31	3.34	*S* < *R*
	**h**	6.02	1.34	4.32	*S* < *R*
***β*^2^-2**	**b**	2.15	1.38	2.36	*-*
	**e**	6.94	1.35	3.85	-
	**g**	3.19	1.40	3.88	*-*
	**h**	7.11	1.35	3.67	*-*
***β*^3^-2**	**b**	2.96	1.36	3.71	*-*
	**e**	5.55	1.42	5.35	*-*
	**g**	2.71	1.35	3.29	*-*
	**h**	5.67	1.58	5.29	*-*
***β*^2^-3**	**b**	3.87	1.62	5.89	*S* < *R*
	**e**	6.04	1.68	6.90	*S* < *R*
	**g**	2.97	1.64	6.19	*S* < *R*
	**h**	6.79	1.53	6.81	*S* < *R*
***β*^3^-3**	**b**	2.75	1.62	3.25	*-*
	**e**	4.74	1.50	3.92	-
	**g**	2.44	1.46	4.03	-
	**h**	4.81	1.49	4.19	-
***β*^2^-4**	**g**	5.60	1.08	0.98	-
	**h**	10.47	1.10	1.07	*-*
***β*^3^-4**	**g**	4.92	1.31	3.73	*S* < *R*
	**h**	9.62	1.25	3.38	*S* < *R*
***β*^2^-5**	**b**	4.57	1.12	1.59	*-*
	**e**	7.70	1.14	2.19	*-*
	**g**	4.27	1.12	1.59	*-*
	**h**	8.01	1.16	1.84	*-*
***β*^3^-5**	**b**	3.91	1.35	3.64	*S* < *R*
	**e**	6.39	1.33	2.80	*S* < *R*
	**g**	3.59	1.36	3.43	*S* < *R*
	**h**	6.64	1.34	3.28	*S* < *R*
***β*^2^-6**	**b**	6.25	1.13	1.59	*-*
	**e**	9.29	1.16	2.24	*-*
	**g**	4.92	1.12	1.56	*-*
	**h**	9.76	1.16	1.92	*-*
***β*^3^-6**	**b**	5.82	1.35	3.56	*S* < *R*
	**e**	8.25	1.30	3.13	*S* < *R*
	**g**	4.39	1.35	3.72	*S* < *R*
	**h**	8.66	1.29	3.00	*S* < *R*

Notes: Chromatographic conditions: column, Chiralpak ZWIX(−)™; mobile phase, **b**, MeOH/MeCN (50/50 *v/v*) containing 25 mM PRA and 50 mM AcOH, **e**, MeOH/MeCN (50/50 *v/v*) containing 25 mM TPRA and 50 mM AcOH, **g**, MeOH/MeCN (50/50 *v/v*) containing 25 mM BA and 50 mM AcOH, **h**, MeOH/MeCN (50/50 *v/v*) containing 25 mM TBA and 50 mM AcOH ; flow rate 0.6 mL·min^−1^; 215, 230 nm and corona detection.

### 2.2. Structure-Retention (Selectivity) Relationship

The position of the substitution in the isobaric enantiomers influences the retention behaviour substantially. On both columns, *β*^2^-amino acids were generally more strongly retained than *β*^3^-amino acids (see earlier). As concerns selectivity, the *α* values for alkyl-substituted analogues on ZWIX(+)™ were slightly larger for the *β*^2^-amino acids, while on ZWIX(−)™ the *α* values were higher for *β*^3^-**1** and for *β*^3^-**2** in the mobile phases containing TPRA or TBA. Of the three alkyl-substituted analogues in the cases of *β*^2^-**3** and *β*^3^-**3**, the retention factors were smaller than for *β*^2^-**1**, *β*^3^-**1**, *β*^2^-**2** and *β*^3^-**2**. With a few exceptions, the smaller retention factors of *β*^2^-**3** and *β*^3^-**3** were associated with a higher *α* on both columns. The results show that in general the *R_S_* values were higher on the ZWIX(−)™ CSP, and *β*^2^-**3** exhibited the highest *R_S_* values on both ZWIX(+)™ and ZWIX(−)™. The presence of the branched 2-propyl group in analyte **3** may have a special effect in chiral recognition.

**Figure 5:**
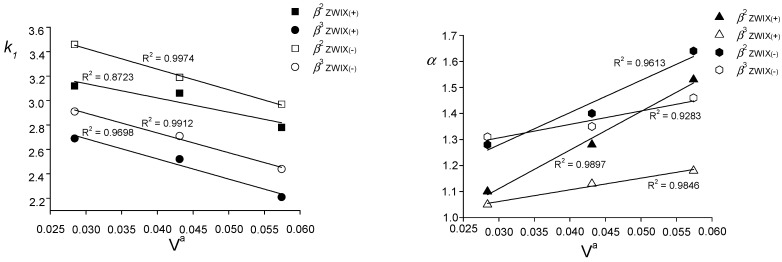
Dependence of retention factors and separation factors of *β*^2^-**1**, *β*^3^-**1**, *β*^2^-**2**, *β*^3^-**2**, *β*^2^-**3** and *β*^3^-**3** on the Meyer substituent parameter (*V^a^*). Chromatographic conditions: column, Chiralpak ZWIX(+)™ and ZWIX(−)™; mobile phase, **g**, MeOH/MeCN (50/50 *v/v*) containing 25 mM BA and 50 mM AcOH; flow rate, 0.6 mL·min^−1^; detection 215 and 230 nm or corona detector; temperature, 25 °C.

In order to determine the specific structural effects of the alkyl substituents in the *β*^2^- and *β*^3^-amino acids on the chromatographic data such as *k*_1_ and *α*, we investigated the volume in the anchor sphere of the substituents (*V^a^*). According to Meyer, the steric effect of a substituent on the reaction rate is characterized by the size-descriptor of the molecule, *V^a^* (Meyer parameter) [41]. For the ZWIX(+)^TM^ and ZWIX(−)^TM^ columns, at a constant mobile phase composition of MeOH/MeCN (50/50 *v*/*v*) containing 25 mM BA and 50 mM AcOH, the chromatographic parameters *k*_1_ and *α* exhibited a strong correlation with *V^a^* (Figure 5). The fits were based on the least-squares method, and for *k*_1_ and *α* good correlations were generally found on both columns. The data in Figure 5 reveal that the retention factors depended strongly on the volume of the alkyl group: a bulkier substituent to some extent inhibited the overall interaction with the SO, and the retention decreased. However, the difference in the interactions of the two enantiomers with the SO differed appreciably, resulting in improved chiral recognition. It may be stated that, besides the position and bulkiness of the substituents, the steric effect strongly influenced the retention (and chiral discrimination) of *β*-amino acid analogues.

Analytes **4**–**6** possess an aromatic ring, which leads to the physical and chemical properties of the compounds being slightly different from those for analytes **1**–**3**. The presence of an aromatic ring favours the π-π interactions which may take place between the molecule and the aromatic ring of the CSP. The data in Table 1 and Table 2 and Figure 3 on the *β*^2^- and *β*^3^-amino acids containing an aromatic side-chain indicate that the *k*_1_ values were higher than those for the amino acids with an alkyl side-chain. Some of the highest *k*_1_ values were obtained in the cases of *β*^2^-**4** and *β*^3^-**4**, which contain a naphthalene ring. Apart from the nature of the base modifiers and columns, the presence of the -Cl group in *β*^2^-**6** and *β*^3^-**6** may improve the interaction with the SO through the H-bonding, which was manifested in higher *k*_1_ values than those of *β*^2^-**5** and *β*^3^-**5** containing a -CH_3_ group.

On the other hand, higher retention was not accompanied by improved chiral recognition: lower *α* values were observed on both columns for the aromatic amino acids than for the analogues containing an alkyl side-chain [exceptions were *β*^3^-**5** and *β*^3^-**6** on ZWIX(−)^TM^; *α* > 1.3]. However, for analytes bearing an aromatic ring, baseline separation was usually attained on ZWIX(−)™ (*R_S_* > 1.5; an exception was *β*^2^-**4**), while *β*^2^-**4** in eluent **h** and *β*^3^-**6** demonstrated baseline or near baseline separation on ZWIX(+)™ (*R_S_* > 1.3).

**Figure 6:**
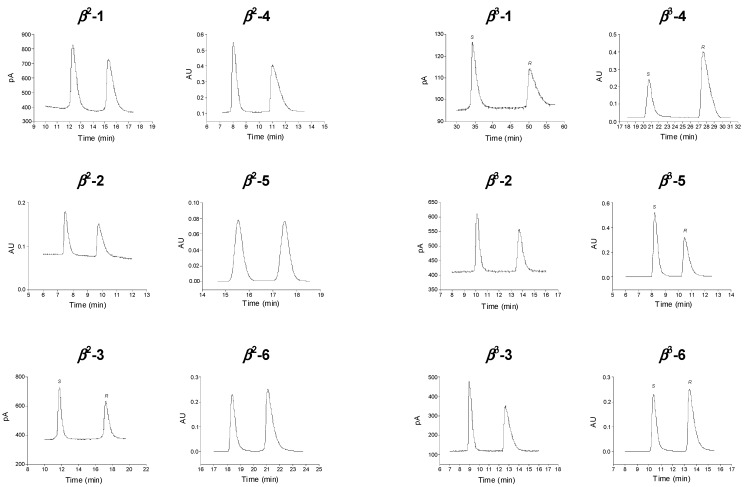
Selected chromatograms for the enantioseparation of *β*^2^- and *β*^3^-amino acids. Chromatographic conditions: column, ZWIX(+) for analyte *β*^2^-**4**, ZWIX(−) for analytes *β*^2^-**1**, *β*^3^-**1**, *β*^2^-**2**, *β*^3^-**2**, *β*^2^-**3**, *β*^3^-**3**, *β*^3^-**4**, *β*^2^-**5**, *β*^3^-**5**, *β*^2^-**6** and *β*^3^-**6**; mobile phase, MeOH/MeCN (50/50 *v/v*) containing 25 mM TPRA and 50 mM AcOH for analytes *β*^2^-**1**, *β*^3^-**2**, *β*^2^-**3**, *β*^2^-**5**, *β*^2^-**6** and *β*^3^-**6**, MeOH/MeCN (25/75 *v/v*) containing 25 mM TPRA and 50 mM AcOH for analyte *β*^3^-**1**, MeOH/MeCN (50/50 *v/v*) containing 25 mM BA and 50 mM AcOH for analytes *β*^2^-**2** and *β*^3^-**5**, MeOH/MeCN (50/50 *v/v*) containing 25 mM TBA and 50 mM AcOH for analytes *β*^3^-**3** and *β*^2^-**4**, MeOH/MeCN (25/75 *v/v*) containing 25 mM PRA and 50 mM AcOH for analyte *β*^3^-**4**; flow rate, 0.6 mL·min^−1^; detection 215 and 230 nm or corona detector; temperature, 25 °C.

The elution sequence was determined in some cases. The nature of the base modifiers did not affect the elution sequence. On ZWIX(+)^TM^, the elution sequence was *R* < *S*, while on ZWIX(−)^TM^ it was *S* < *R*. This indicates that the SOs behave pseudo-enantiomerically and that the binding pocket around C8 and C9 influences predominantly the molecular recognition events. The reversal of the elution sequence on *Cinchona* alkaloid-based CSPs is advantageous as regards the separation of the minor component in the presence of the major one. Selected chromatograms depicting baseline resolution are presented in Figure 6.

### 2.3. Effects of Temperature and Thermodynamic Parameters

In order to investigate the effects of temperature, a variable-temperature study was carried out on ZWIX(+)™ and ZWIX(−)™ columns over the temperature range 10–50 °C (in 10 °C increments). For the *β*^2^- and *β*^3^-amino acids (analytes **1**, **3**, **4** and **6**), the measurements were made with the mobile phase system MeOH/MeCN (50/50 *v/v*) containing 25 mM PRA and 50 mM AcOH. The chromatographic parameters *k*_1_ and *α* decreased or did not change significantly with increasing temperature, except in the case of *β*^2^-**4** on the ZWIX(−)^TM^ CSP, where *α* (and *R_S_*) increased slightly with increasing temperature (Supplementary Material, Tables S3 and S4). The *R_S_* values usually decreased with increasing temperature, while for *β*^3^-**1**, *β*^3^-**4**, *β*^2^-**6** and *β*^3^-**6** on ZWIX(−)^TM^, a maximum curve was observed with increase of temperature. At higher temperatures, *R_S_* decreased due to the smaller *α*, while the decrease in *R_S_* at lower temperatures may be explained by the dominating effect of the decreased column efficiency. Since the effect of temperature on the separation was more complex, an extensive study dealing with the thermodynamics of enantiomer separation was carried out.

The thermodynamic parameters were obtained from van’t Hoff plots (Equation (2)); the changes in standard enthalpy ∆(∆*H*°) and entropy ∆(∆*S*°) derived from ln *α* *vs.* 1/*T* plots are presented in Table 3. The ∆(∆*H*°) values ranged from −9.7 to −0.4 kJ·mol^−1^ on ZWIX(+)™, and from −7.7 to +0.3 kJ·mol^−1^ on ZWIX(−)™. The *ß*^3^-amino acids usually exhibited larger −∆(∆*H*°) values than those of the *β*^2^-amino acids on both CSPs [*β*^2^-**4** was not separable on ZWIX(+)™]. For *β*^2^-**1** and all the *β*^3^-amino acid analogues, the −∆(∆*H*°) values were larger on ZWIX(−)^TM^ while for *β*^2^-**3** and *β*^2^-**6** they were larger on ZWIX(+)^TM^. The interactions of *β*^3^-**3** and *β*^2^-**3** with the ZWIX(+)™ and ZWIX(−)™ CSP were characterized by the highest negative ∆(∆*H*°) and ∆(∆*S*°) values, indicating the importance of steric effects in the stabilization of the SO-SA complex.

Under the conditions where *∆*(*∆H°*) has negative values, *∆*(*∆S°*) was also negative, and positive *∆*(*∆H°*) was accompanied by positive *∆*(*∆S*°) [*β*^2^-**4** on ZWIX(−)™]. The trends in the change in *∆*(*∆S*°) showed that *β*^3^-amino acids exhibited larger −*∆*(*∆S*°) values than did the *β*^2^-amino acids. In general, it was also observed that these values were higher for the amino acids containing an alkyl side-chain than for those with an aromatic side-chain (Table 3). The ln *α* *vs. 1/T* plots were in most cases characterized by linear fits, but for *β*^3^-**3** on ZWIX(+)™ the ln *α* *vs. 1/T* plot could be divided into two linear regions, which means that the linear van’t Hoff plots reflect different overall binding situations in limited temperature ranges. (In these cases, Table 3 presents values calculated for the two temperature ranges independently; *β*^2^-**6** on ZWIX(+)™ exhibited separation in the temperature range 10–30 °C. In the temperature range 10–30 °C for *β*^3^-**3** on ZWIX(+)™, the separations involved relatively small −*∆*(*∆H*°) and larger −*T∆*(*∆S*°) values, *i.e.*, a larger contribution of entropy to the enantioseparation was observed in this temperature region. For analyte *β*^2^-**4** on ZWIX(−)™, both *∆*(*∆S*°) and *∆*(*∆H*°) were positive, indicating an entropically driven separation.

The thermodynamic parameter −*∆*(*∆G*°)_298_ suggests that, both on ZWIX(+)™ and on ZWIX(−)™ for *β*^2^-**3** and *β*^3^-**3**, the interaction with the SO was more efficient, as reflected by the largest −*∆*(*∆G*°) values. It can be concluded from the −*∆*(*∆H*°) and −*T∆*(*∆S°*) data for all the analytes that the enantioresolution is predominantly enthalpically driven, and the selectivity decreases with increasing temperature. For analyte *β*^2^-**4** on the ZWIX(−)™, the −*T∆*(*∆S°*) value were more positive than the *∆*(*∆H*°), indicating an entropically driven separation. The −*∆*(*∆G*°)_298_ values were generally slightly larger on ZWIX(−)™ than on ZWIX(+)™, which is in accordance with results obtained for unusual *β*-amino acids [39,40].

**Table 3:** Thermodynamic parameters, *∆*(*∆H°*), *∆*(*∆S°*), Tx∆(∆*S°*), *∆*(*∆G°*) and correlation coefficients (*R*^2^) of *β*^2^- and *β*^3^-amino acids on ZWIX(+)™ and ZWIX(−)™ column.

Analyte	Column	−∆(∆*H°*) (kJ·mol^−1^)	−∆(∆*S°*) (J·mol^−1^·K^−1^)	Corr. coeff. (*R*^2^)	−Tx∆(∆*S°*)_298K_ (kJ·mol^−1^)	−∆(∆*G°*)_298K_ (kJ·mol^−1^)
*β*^2^-**1**	ZWIX(+)™	0.4	0.6	0.9956	0.2	0.2
	ZWIX(−)™	0.9	1.0	0.9914	0.3	0.6
*β*^3^-**1**	ZWIX(+)™	0.8	2.3	0.9932	0.7	0.1
	ZWIX(−)™	2.9	7.3	0.9926	2.2	0.7
*β*^2^-**3**	ZWIX(+)™	3.8	9.0	0.9945	2.7	1.1
	ZWIX(−)™	3.3	7.1	0.9965	2.1	1.2
*β*^3^-**3**	ZWIX(+)™ *	3.9 *	9.7 *	0.9935	2.9 *	1.0 *
	ZWIX(+)™ **	9.7 **	29.1 **	0.9996	8.7 **	1.0 **
	ZWIX(−)™	7.7	21.7	0.9953	6.5	1.2
*β*^2^-**4**	ZWIX(+)™	-	-	-	-	-
	ZWIX(−)™	−0.3	−1.7	0.9918	−0.5	0.2
*β*^3^-**4**	ZWIX(+)™	0.4	0.5	0.9945	0.2	0.2
	ZWIX(−)™	1.8	4.0	0.9908	1.2	0.6
*β*^2^-**6**	ZWIX(+)™ *	0.6 *	1.9 *	0.9998	0.6 *	0.0 *
	ZWIX(−)™	0.3	0.1	0.9972	0.04	0.3
*β*^3^-**6**	ZWIX(+)™	0.5	0.5	0.9936	0.1	0.4
	ZWIX(−)™	1.9	3.8	0.9962	1.1	0.8

Notes: Chromatographic conditions: column, Chiralpak ZWIX(+)™ and ZWIX(−)™; mobile phase, **b**, MeOH/MeCN (50/50 *v/v*) containing 25 mM PRA and 50 mM AcOH; flow rate 0.6 mL·min^−1^; 215 and 230 nm detection; *R*^2^, correlation coefficient of van’t Hoff plot, ln *α* − 1/*T* curves, * temperature range: 10–30 °C, ** temperature range 30–50 °C.

## 3. Experimental

### 3.1. Chemicals and Syntheses

Four-step syntheses were applied for the production of racemic 3-amino-2-methylpropionic acid (*β*^2^-**1**), 2-aminomethylbutanoic acid (*β*^2^-**2**), 2-aminomethyl-3-methylbutanoic acid (*β*^2^-**3**), 3-amino-2- (naphthalen-2-ylmethyl)propionic acid (*β*^2^-**4**), 3-amino-2-(4-methylbenzyl)propionic acid (*β*^2^-**5**) and 3-amino-2-(4-chlorobenzyl)propionic acid (*β*^2^-**6**) (Figure 1). In the first step, the starting material methyl cyanoacetate was either alkylated or condensed with an aromatic aldehyde [42,43,44], and in the next step it was reduced in the presence of di-*tert*-butyl dicarbonate [45]. Upon deprotection of the methyl ester by hydrolysis and the Boc-carbamate by acidolysis, a racemic mixture of free *β*^2^-amino acids was obtained [46]. Enantiomer (*S*)***-****β*^2^-**3** was a generous gift from Prof. D. Tourwé (Vrije Universiteit Brussels, Brussels, Belgium).

Racemic 3-aminobutanoic acid (*β*^3^-**1**) and 3-aminopentanoic acid (*β*^3^-**2**) (Figure 1) were prepared from the corresponding *α,β*-unsaturated acids by benzylamine addition and subsequent debenzylation of the products with 20% metallic palladium on charcoal in a hydrogen atmosphere [47,48]. (*R*)-*β*^3^-**1**) was prepared by the same method, but (*R*)-(+)-*α*-methylbenzylamine was used in the addition step instead of benzylamine [49]. 3-Amino-4-methylpentanoic acid (*β*^3^-**3**), 3-amino-4-(naphthalen-2-yl)butanoic acid (*β*^3^-**4**), 3-amino-4-(4-methylphenyl)butanoic acid (*β*^3^-**5**) and 3-amino-4-(4-chlorophenyl)butanoic acid (*β*^3^-**6**) were synthetized from the corresponding aldehydes by a modified procedure of Rodionov and Malevinskaya: the aldehydes were condensed with an equimolar amount of malonic acid in refluxing 96% ethanol in the presence of two equivalents of ammonium acetate [50,51].

Acetonitrile (MeCN), methanol (MeOH) of HPLC grade, propylamine (PRA), tripropylamine (TPRA), butylamine (BA), tributylamine (TBA), glacial acetic acid (AcOH) and other reagents of analytical reagent grade were from VWR International (Radnor, PA, USA). The Milli-Q water was further purified by filtration on a 0.45-μm filter, type HV, Millipore (Molsheim, France).

### 3.2. Apparatus and Chromatography

The apparatus for chromatography comprised a Waters Breeze system consisting of a 1525 binary pump, a 487 dual-channel absorbance detector, a 717 plus autosampler and Empower 2 data manager software (Waters Chromatography, Milford, MA, USA). The columns were thermostated in a Spark Mistral column thermostat (Spark Holland, Emmen, The Netherlands). The alternative 1100 Series HPLC system consisted of a solvent degasser, a pump, an autosampler, a column thermostat, a multi wavelength UV-VIS detector (all from Agilent Technologies, Waldbronn, Germany), and a CORONA charged aerosol detector from ESA Biosciences, Inc. (Chelmsford, MA, USA). Data acquisition and analysis were carried out with ChemStation chromatographic data manager software from Agilent Technologies. The precision of temperature adjustment was ±0.1 °C. The Chiralpak ZWIX(+)™ and ZWIX(−)™ columns (150 × 3.0 mm I.D., 3-μm particle size for both columns) were provided by Chiral Technologies Europe (Illkirch, France).

Chromatography was performed in isocratic mode at a flow rate of 0.6 mL·min^−1^; the column temperature was varied in 10 °C increments between 10 and 50 °C. Detection was accomplished by UV and corona discharge detection. The void volume of the columns (*t*_0_) was determined with acetone dissolved in MeOH. Solutions of analytes were made in MeOH in the concentration range 0.5–1.0 mg·mL^−1^ and further diluted with the mobile phase.

## 4. Conclusions

The enantioseparation of a series of isobaric *β*^2^- and *β*^3^-homoamino acid analogues was investigated by using Chiralpak ZWIX(+)™ and ZWIX(−)™ columns, containing quinine- or quinidine-based zwitterionic selectors. The separations were accomplished in PIM by using MeOH/MeCN mobile phases with different compositions, containing PRA, TPRA, BA or TBA as base and AcOH as acid additive. The chromatographic parameters depended on the mobile phase composition, the nature and concentrations of the mobile phase additives and temperature. Baseline resolution was achieved in all cases. Of the two ampholytic columns, the quinidine-based ZWIX(−)^TM^ appeared more suitable for the enantioseparation of the investigated *β*-amino acids, and both the ZWIX(+)™ and ZWIX(−)™ columns exhibited better separation performances for *β*^2^- than for *β*^3^-amino acids.

The values of the thermodynamic parameters, such as the changes in enthalpy, Δ(*∆H°*), entropy, Δ(*∆S°*), and Gibbs energy, Δ(*∆G°*), depended on the structures of the analytes and on the chiral selectors employed.

The elution sequence was determined in some cases and revealed that these CSPs behave pseudo-enantiomerically to each other, leading to a reversal of the elution sequence on the quinine-based ZWIX(+)™ and on the quinidine-based ZWIX(−)™ CSPs. This is advantageous from the aspect of the chiral separation of a minor component in the presence of a major one.

## Supplementary Materials

Supplementary materials can be accessed at: http://www.mdpi.com/1420-3049/20/01/0070/s1.
