# Supplementary material for: Comparison of the Separation Performances of Cinchona Alkaloid-Based Zwitterionic Stationary Phases in the Enantioseparation of β2- and β3-Amino Acids
Source: Molecules. 2014 Dec 23;20(1):70–87. doi: 10.3390/molecules20010070 (PMC6272689; doi:10.3390/molecules20010070)
Supplement: Supplementary file 1 [file molecules-20-00070-s001.pdf]

Supplementary Materials

Table S1. Chromatographic data, retention factor (k), selectivity factor (α), resolution (R_S) and elution sequence of β^2- and β^3-amino acids on ZWIX(+)™ column.

Compound	Eluent	k_1	k_2	α	R_S	*Elution sequence*
β^2-**1**	**a**	2.42	2.66	1.10	0.80	-
	**b**	4.00	4.39	1.10	1.09	-
	**c**	7.21	8.05	1.12	0.86	-
	**d**	2.78	3.00	1.08	0.35	-
	**e**	5.64	6.06	1.07	0.55	-
	**f**	14.45	16.02	1.11	<0.20	-
β^3-**1**	**a**	2.18	2.18	1.00	0.00	-
	**b**	3.30	3.52	1.07	0.70	-
	**c**	5.93	6.99	1.18	0.52	$R < S$
	**d**	2.45	2.45	1.00	0.00	-
	**e**	4.76	5.07	1.07	0.71	$R < S$
	**f**	12.60	14.34	1.13	0.33	-
β^2-**4**	**a**	4.64	4.64	1.00	0.00	-
	**b**	5.48	5.48	1.00	0.00	-
	**c**	11.98	12.33	1.03	0.30	-
	**d**	5.64	5.64	1.00	0.00	-
	**e**	9.43	9.43	1.00	0.00	-
	**f**	22.92	24.82	1.08	<0.20	-
β^3-**4**	**a**	4.12	4.12	1.00	0.00	-
	**b**	5.20	5.73	1.10	1.06	$R < S$
	**c**	9.00	10.60	1.18	1.85	$R < S$
	**d**	5.11	5.11	1.00	0.00	-
	**e**	7.77	8.24	1.06	0.37	$R < S$
	**f**	20.15	22.60	1.12	1.06	$R < S$

Notes: Chromatographic conditions: column, Chiralpak ZWIX(+)™; mobile phase, **a**, MeOH/MeCN (75/25 *v/v*) containing 25 mM PRA and 50 mM AcOH, **b**, MeOH/MeCN (50/50 *v/v*) containing 25 mM PRA and 50 mM AcOH, **c**, MeOH/MeCN (25/75 *v/v*) containing 25 mM PRA and 50 mM AcOH, **d**, MeOH/MeCN (75/25 *v/v*) containing 25 mM TPRA and 50 mM AcOH, **e**, MeOH/MeCN (50/50 *v/v*) containing 25 mM TPRA and 50 mM AcOH, **f**, MeOH/MeCN (25/75 *v/v*) containing 25 mM TPRA and 50 mM AcOH; flow rate 0.6 $mL \cdot min^{-1}$; 215, 230 nm and corona detection.

Table S2. Chromatographic data, retention factor (*k*), selectivity factor (*α*), resolution (R_S) and elution sequence of *β*[2]- and *β*[3]-amino acids on ZWIX(−)™ column.

Compound	Eluent	k_1	k_2	α	R_S	*Elution sequence*
*β*²-**1**	**a**	2.95	3.62	1.23	1.78	-
	**b**	4.22	5.35	1.27	2.10	-
	**c**	10.04	12.95	1.29	1.66	-
	**d**	3.44	4.19	1.22	1.21	-
	**e**	6.95	8.91	1.28	3.40	-
	**f**	24.71	33.01	1.34	3.05	-
*β*³-**1**	**a**	2.73	3.32	1.21	1.75	*S* < *R*
	**b**	3.72	4.90	1.32	2.29	*S* < *R*
	**c**	8.83	10.92	1.24	1.71	*S* < *R*
	**d**	2.98	3.58	1.20	1.43	*S* < *R*
	**e**	5.88	7.99	1.36	4.51	*S* < *R*
	**f**	19.21	28.64	1.49	4.80	*S* < *R*
*β*²-**4**	**a**	5.59	5.85	1.05	0.36	-
	**b**	6.23	6.73	1.08	0.86	-
	**c**	15.88	17.83	1.12	1.75	-
	**d**	6.52	6.87	1.05	0.50	-
	**e**	11.51	12.52	1.09	1.25	-
	**f**	31.92	36.00	1.13	1.63	-
*β*³-**4**	**a**	5.31	6.26	1.18	2.00	*S* < *R*
	**b**	6.27	8.02	1.28	3.20	*S* < *R*
	**c**	12.85	17.45	1.36	4.21	*S* < *R*
	**d**	6.14	6.99	1.14	1.60	*S* < *R*
	**e**	10.34	12.91	1.25	2.95	*S* < *R*
	**f**	26.68	35.39	1.33	3.50	*S* < *R*

Notes: Chromatographic conditions: column, Chiralpak ZWIX(−)™; mobile phase, **a**, MeOH/MeCN (75/25 *v/v*) containing 25 mM PRA and 50 mM AcOH, **b**, MeOH/MeCN (50/50 *v/v*) containing 25 mM PRA and 50 mM AcOH, **c**, MeOH/MeCN (25/75 *v/v*) containing 25 mM PRA and 50 mM AcOH, **d**, MeOH/MeCN (75/25 *v/v*) containing 25 mM TPRA and 50 mM AcOH, **e**, MeOH/MeCN (50/50 *v/v*) containing 25 mM TPRA and 50 mM AcOH, **f**, MeOH/MeCN (25/75 *v/v*) containing 25 mM TPRA and 50 mM AcOH; flow rate 0.6 mL·min^{-1}; 215, 230 nm or corona detection.

Table S3. Temperature dependence of retention factor of first eluting enantiomer (k_1), second eluting enantiomer (k_2), separation factor (α) and resolution (R_S) of β^2- and β^3-amino acids on ZWIX(+)™ column.

Compound	*k, α, R_S*	Temperature (°C)					*Elution sequence*
		10	20	30	40	50	
**β^2-1**	k_1	4.95	4.41	3.35	2.74	2.23	-
	k_2	5.49	4.85	3.67	2.98	2.42	
	α	1.11	1.10	1.10	1.09	1.09	
	R_S	1.21	1.16	1.05	0.98	0.92	
**β^3-1**	k_1	4.34	3.82	2.97	2.31	1.97	$R < S$
	k_2	4.71	4.09	3.15	2.42	2.04	
	α	1.09	1.07	1.06	1.05	1.04	
	R_S	1.35	0.78	0.67	0.44	0.20	
**β^2-3**	k_1	4.04	3.80	3.02	2.47	2.09	$R < S$
	k_2	6.68	6.04	4.52	3.52	2.85	
	α	1.65	1.59	1.50	1.43	1.36	
	R_S	6.72	5.93	5.20	4.52	3.95	
**β^3-3**	k_1	2.46	2.38	2.11	1.83	1.57	-
	k_2	3.96	3.60	3.05	2.35	1.79	
	α	1.61	1.51	1.44	1.28	1.14	
	R_S	2.45	2.04	1.71	1.66	1.36	
**β^2-4**	k_1	7.00	6.02	5.01	4.48	3.88	-
	k_2	7.00	6.02	5.01	4.48	3.88	
	α	1.00	1.00	1.00	1.00	1.00	
	R_S	0.00	0.00	0.00	0.00	0.00	
**β^3-4**	k_1	7.26	6.11	4.78	3.42	2.86	$R < S$
	k_2	8.01	6.71	5.23	3.72	3.09	
	α	1.10	1.10	1.09	1.09	1.08	
	R_S	1.12	1.11	1.06	0.98	0.86	
**β^2-6**	k_1	7.06	6.32	4.97	3.98	3.30	-
	k_2	7.29	6.48	5.05	3.98	3.30	
	α	1.03	1.02	1.02	1.00	1.00	
	R_S	0.20	<0.20	<0.20	0.00	0.00	
**β^3-6**	k_1	6.38	5.39	4.17	3.12	2.47	$R < S$
	k_2	7.40	6.20	4.78	3.55	2.79	
	α	1.16	1.15	1.15	1.14	1.13	
	R_S	1.95	1.83	1.68	1.59	1.43	

Notes: Chromatographic conditions: column, Chiralpak ZWIX(+)™; mobile phase, **b**, MeOH/MeCN (50/50 *v/v*) containing 25 mM PRA and 50 mM AcOH; flow rate 0.6 mL·min^{-1}; detection, 215 and 230 nm or corona detection.

Table S4. Temperature dependence of retention factor of first eluting enantiomer (k_1), second eluting enantiomer (k_2), separation factor (α) and resolution (R_S) of β^2- and β^3-amino acids on ZWIX(−)™ column.

Compound	*k, α, R_S*	Temperature (°C)					*Elution sequence*
		10	20	30	40	50	
β^2-**1**	k_1	5.04	4.71	3.85	3.31	2.78	-
	k_2	6.53	6.00	4.86	4.12	3.43	
	α	1.29	1.28	1.26	1.24	1.24	
	R_S	2.40	2.26	2.44	2.19	2.07	
β^3-**1**	k_1	4.44	4.06	3.32	2.74	2.19	$S < R$
	k_2	6.21	5.47	4.32	3.44	2.63	
	α	1.40	1.35	1.30	1.25	1.20	
	R_S	2.24	3.05	2.60	2.32	1.85	
β^2-**3**	k_1	4.16	4.08	3.66	2.95	2.50	$S < R$
	k_2	7.26	6.74	5.77	4.47	3.67	
	α	1.74	1.65	1.58	1.51	1.47	
	R_S	6.61	6.15	5.87	5.32	4.80	
β^3-**3**	k_1	3.02	2.88	2.64	2.24	1.90	-
	k_2	5.73	5.03	4.09	3.16	2.42	
	α	1.90	1.75	1.55	1.41	1.27	
	R_S	3.98	3.77	3.25	2.89	2.59	
β^2-**4**	k_1	7.04	6.47	5.85	5.28	4.72	-
	k_2	7.58	7.00	6.35	5.76	5.16	
	α	1.08	1.08	1.09	1.09	1.09	
	R_S	1.00	0.95	1.18	1.18	1.20	
β^3-**4**	k_1	7.99	7.17	5.99	4.60	3.65	$S < R$
	k_2	10.67	9.39	7.57	5.72	4.45	
	α	1.34	1.31	1.27	1.24	1.22	
	R_S	3.49	3.65	3.41	3.10	2.86	
β^2-**6**	k_1	7.39	6.93	5.65	4.73	3.94	-
	k_2	8.38	7.82	6.34	5.30	4.39	
	α	1.13	1.13	1.12	1.12	1.11	
	R_S	1.42	1.47	1.53	1.50	1.38	
β^3-**6**	k_1	7.05	6.47	5.00	4.13	3.30	$S < R$
	k_2	9.93	8.89	6.73	5.40	4.22	
	α	1.41	1.37	1.35	1.31	1.28	
	R_S	3.55	3.94	3.70	3.33	3.06	

Notes: Chromatographic conditions: column, Chiralpak ZWIX(−)™; mobile phase, **b**, MeOH/MeCN (50/50 *v/v*) containing 25 mM PRA and 50 mM AcOH; flow rate 0.6 mL·min^{-1}; 215 and 230 nm or corona detection.
